# Indirect consequences of extreme weather and climate events and their associations with physical health in coastal Bangladesh: a cross-sectional study

**DOI:** 10.3402/gha.v8.29016

**Published:** 2015-10-19

**Authors:** Dominik Beier, Patrick Brzoska, Mobarak Hossain Khan

**Affiliations:** 1 Department of Anesthesiology and Surgical Intensive Care Medicine, Medical Faculty Mannheim, Heidelberg University, Mannheim, Germany; 2 Unit of Epidemiology, Institute of Sociology, Faculty of Behavioral and Social Sciences, Chemnitz University of Technology, Chemnitz, Germany; 3 Department of Public Health Medicine, School of Public Health, Bielefeld University, Bielefeld, Germany

**Keywords:** Bangladesh, extreme weather and climate events, physical health, climate change, coastal flooding

## Abstract

**Background:**

Bangladesh is one of the countries in the world which is most prone to natural disasters. The overall situation is expected to worsen, since extreme weather and climate events (EWCE) are likely to increase in both frequency and intensity. Indirect consequences caused in the events’ aftermath widen the range of possible adverse health outcomes.

**Objective:**

To assess the association of indirect consequences of EWCE and physical health.

**Design:**

We used recent cross-sectional self-reported data from 16 coastal villages in Bangladesh. A total of 980 households were surveyed using a structured questionnaire. The outcome of physical health was categorized into three groups, reflecting the severity of reported diseases by the respective source of treatment as a proxy variable (hospital/clinic for severe disease, other source/no treatment for moderate disease, and no disease). The final statistical analysis was conducted using multinomial logistic regression.

**Results:**

Severe diseases were significantly associated with drinking water from open sources [odds ratio (OR): 4.26, 95% confidence interval (CI): 2.25–8.09] and tube wells (OR: 2.39, 95% CI: 1.43–4.01), moderate harm by river erosion (OR: 6.24, 95% CI: 2.76–14.11), food scarcity (OR: 1.98, 95% CI: 1.16–3.40), and the perception of increased employment problems (OR: 2.19, 95% CI: 1.18–4.07). Moderate diseases were significantly associated with moderate harm by river erosion (OR: 2.65, 95% CI: 1.28–5.48) and fully experienced food scarcity (OR: 1.75, 95% CI: 1.16–2.63). For both categories, women and the elderly had higher chances for diseases.

**Conclusions:**

Indirect consequences of EWCE were found to be associated with adverse health outcomes. Basic needs such as drinking water, food production, and employment opportunities are particularly likely to become threatened by EWCE and, thus, may lead to a higher likelihood of ill-health. Intervention strategies should concentrate on protection and provision of basic needs such as safe drinking water and food in the aftermath of an event.

Human-induced climate change has become more and more evident in the recent decades. It is caused mainly by the accumulation of greenhouse gases, and several indicators such as ongoing global warming or rise in sea level have been comprehensively studied and documented by climatologists and the Intergovernmental Panel on Climate Change (IPCC) (1). As a consequence, it is very likely that certain weather and climate events increase in both frequency and intensity (1, 2). Although most of these phenomena can be observed worldwide, it is mainly the developing countries in the tropical and subtropical regions which have to face the consequences of these events to a greater extent (2, 3). Many of these societies, thus, have a greater vulnerability to extreme weather and climate events (EWCE), which in this case isa function of exposure to EWCE, sensitivity of population's health and its influencing factors, and adaptation measures to reduce the health-related burden (4). The impact of EWCE on health is thereby a crucial point. As the IPCC states, most of the risks that occur due to a high vulnerability of exposed societies or systems are related to health, especially coastal areas are more at risk (5). Health risks can occur via direct or indirect consequences. Indirect consequences of EWCE are likely to widen the range of possible adverse health outcomes in the aftermath of EWCE, in contrast to direct consequences with immediate effects on health (2, 6, 7). Usually, indirect consequences are present in the long term and include changes in the environment or disruptions to ecological systems such as worsening of freshwater quality and changes in the distribution of infectious diseases, and social problems such as food shortages or the loss of sources of income (2, 6, 8). For instance, obvious adverse health outcomes can result because of vector-borne diseases arising from stagnant water after floods or because of contaminated drinking water, all the more when the sewage system is damaged, or because of the loss of property and the necessity to move, which can cause mental disorders (2, 6–9). On the contrary, direct consequences include, for example, injuries and deaths through cyclones or heat waves (6–8).

One of the developing countries being particularly threatened by EWCE is Bangladesh because of its high vulnerability and exposure. With a coastline of nearly 600 km and deltas of great rivers such as the Ganges, Jamuna, and Padma, Bangladesh is naturally exposed to environmental challenges because of its landscape alone. In addition, high population density and growth lead to a larger population at risk for natural calamities with adverse health consequences (6, 8, 10). EWCE-related consequences are, therefore, likely to increase the already high disease burden in Bangladesh, especially in terms of transmission of infectious diseases. Although a notable shift from communicable to non-communicable diseases is evident (11–13), Bangladesh is suffering from a severe double disease burden due to the slow decline of communicable diseases (11).

In terms of concrete events, floods and tropical cyclones are two examples of EWCE that are particularly a threat to Bangladesh (8, 10). Despite this obvious exposure to natural hazards, few studies have been conducted so far with the aim of identifying the impacts of climate change, especially the impacts of emerging EWCE on population health in terms of specific diseases (8, 14). One example of a well-studied disease pattern is diarrheal diseases, which can be influenced by climatic and weather-related indicators (15–17). By analyzing hospital surveillance data of a 7-year period together with meteorological data in Dhaka, the capital of Bangladesh, Hashizume et al. (16) found that non-cholera diarrhea was significantly associated with an increase of river levels because of higher rainfall which eventually led to an increased incidence of diarrhea. Similar results were found in the same study setting for rotavirus infections as one specific cause for non-cholera diarrhea, which was also explained by an increase of river level due to flood water (17). A large proportion of coastal population in Bangladesh uses surface water (e.g. river and pond water) as a source of drinking water (18), through which infectious disease agents can easily be transmitted. Moreover, increase in salt levels in soil water after coastal flooding also poses an additional threat to natural water sources in coastal regions (18). Other than the contamination of drinking water, coastal flooding also has a negative impact on food production, leading to destroyed crop yields in a situation where the demand will grow further because of advancing overall population growth (19).

Altogether, consequences on health associated with EWCE are well-known, and there are some studies that provide data on specific diseases or associated factors (15–17). However, little is known about the perception of climate change of populations at risk, especially regarding indirect consequences. Although Haque et al. (20) studied the perception of climate change and certain health risks among people in Bangladesh in a descriptive study, there still remains some uncertainty about what other factors are actually associated with adverse health outcomes related to EWCE as perceived by the population, especially in terms of indirect consequences. Therefore, the main objective of this study was the assessment of indirect consequences of EWCE and their association with ill-health with a special focus on perceived changes which can be linked to climate change.

## Methods

We used cross-sectional self-reported data for this study. Members of the Department of Public Health Medicine at Bielefeld University, Germany, collected the data in the fall of 2013. The study area is located in the Sarankhola *upazila* (one of 488 administrative regions of Bangladesh and most affected by the cyclones Sidr and Aila in 2007 and 2009, respectively), which is in the Bagerhat district of Khulna division in south Bangladesh. In this *upazila*, 16 out of 44 coastal villages were selected as study sites. The households were chosen via a systematic sampling approach, where every third or fourth household was included, depending on the respective population density. Structured questionnaires were used for the interviews, whereby male adults were approached when possible to gain a balanced proportion of males and females, since most men were usually out for work when survey was conducted. We explained the objectives of the survey and got verbal consent from each participant before starting the interview. Participants were allowed to skip any question and/or quit the interview at any time. We also talked to the local leaders and the head of the government officials in this *upazila* before starting the survey. Finally, the data were analyzed anonymously to maintain confidentiality. Therefore, none of the participants can be linked with the data.

### Sample size

The sample size for the particular analysis was calculated according to the harm inflicted on a household by coastal flooding and/or cyclones. Since our focus was on indirect consequences of EWCE, only those households that were affected by at least one of these events were selected. In the survey, every respondent was asked whether his or her family had been seriously harmed by coastal flooding and cyclone/tornado (possible answers were ‘yes’, ‘moderately yes’, ‘no’, or ‘don't know’). Consequently, only 977 of the 980 households in the original dataset, which were at least moderately harmed by either coastal flooding or cyclones/tornados, were considered for further analysis.

### Dependent variable

The outcome variable was physical health with three categories. This variable was defined in the following manner: First, the respondents were asked whether they had any disease (self-perceived) in the past month prior to the survey (‘yes’, ‘no’). In case of affirmative answer, they were asked whether they went for any treatment for the past disease (‘yes’, ‘no’). If they answered ‘yes’ for treatment, they were finally asked to mention the sources of treatment. Several sources of treatment, for example, hospital/clinics (governmental/non-governmental), pharmacies, or traditional medicines were mentioned. For the present analysis, they were pooled into three categories to reflect the severity of the disease as reported by the respondents: ‘severe disease’ if the respondent sought treatment in any form at a hospital/clinic, ‘moderate disease’ if the respondent sought any other form of treatment (e.g. pharmacy or traditional medicine) or no treatment at all, and ‘no disease’ if the respondent did not suffer from any disease in the past month prior to the survey. This categorization was used since no medical verification of the diseases was possible and also to limit both the recall and information bias, which are typical for self-reported data.

### Independent variables

The survey further contained information regarding possible associated factors. First, important household characteristics such as the source of drinking water (‘tube well’, ‘pond/river/lake’, ‘supply’) and the type of toilet facility (‘slab toilet’, ‘open latrine’, ‘modern toilet’) were included. Second, the harm to the livelihood of the respondent's family by EWCE and associated consequences was reflected by salinity in land, river erosion, and food scarcity, where the respondents mentioned whether they were harmed or not (‘yes’, ‘moderately yes’, ‘no’). Third, the perception of EWCE-related consequences was included to reflect changes that were observed by the respondents in the past few years. Each question regarding waterborne diseases, water logging, loss of houses and animals, loss of agricultural fields, social problems (e.g. robbery, violence), sewerage problems, drinking water availability, employment problems, and sanitation problems were answered by any of the three options: ‘increased’, ‘decreased’, or ‘almost same’. Finally, possible confounding factors were included, namely age, educational level, the main source of family income (as a proxy for the socioeconomic status since the annual income might vary due to seasonal effects), body mass index (BMI), level of physical labor, and smoking status. Age was categorized into groups ranging from 14 to 25, 26 to 35, 36 to 45, 46 to 55, and 56+ years (these age groups were selected to gain approximately equal-sized groups); the categorization of the educational level reflects the educational system in Bangladesh, ranging from levels of no education, 1 to 5 years, 6 to 10 years, and 11+ years; the main source of family income included ‘agriculture’, ‘business’, ‘public service’, ‘day labor’, ‘fishing’, and ‘others’; the levels of BMI were calculated according to WHO criteria (21), ranging from underweight, normal weight, and overweight to obesity; the smoking status identified smokers (‘yes’) and non-smokers (‘no’), while the levels of physical labor were categorized as ‘very high’, ‘high’, ‘so-so’, and ‘low’.

### Statistical analysis

For the statistical analysis, we performed simple to multivariable, multinomial logistic regression analyses. Using bivariable analysis, all selected independent variables were first tested for stochastic independence with the outcome variable by using Pearson's χ^2^ test. Those variables which were found significant in the bivariable analysis (95% confidence) were included in the multinomial logistic regression model. Confounding variables which were significant in the bivariable analysis were forced into the model, whereas all other variables were included by using a backward stepwise procedure so that all non-significant variables would be automatically excluded. Multicollinearity among independent variables was also checked before inserting them into the multivariable model. The analysis was conducted with IBM SPSS Statistics Version 22.

## Results

### Sample description

As presented in Table 1, the sample consisted of an almost equally balanced proportion of males and females who were mostly young, moderately educated, and daily wage laborers. Most respondents had normal weight, while one fourth was underweight. The households predominately used supply water as their main source of drinking water and the traditional slab toilets for defecation.

**Table 1:** Basic characteristics of the respondents and their bivariable associations with severity of disease

Simple analysis		Severity of disease			
	
Variable	Frequency^a^	Severe disease	Moderate disease	No disease	*p*
Physical health	977 (100%)	204 (20.9%)	501 (51.3%)	272 (27.8%)	
Sex					**<0.001**
Male	465 (47.6%)	86 (18.5%)	215 (46.2%)	164 (35.3%)	
Female	512 (52.4%)	118 (23%)	286 (55.9%)	108 (21.1%)	
Age					**0.003**
56 +	161 (16.5%)	38 (23.6%)	97 (60.2%)	26 (16.1%)	
46–55	162 (16.6%)	38 (23.5%)	79 (48.8%)	45 (27.8%)	
36–45	215 (22%)	56 (26%)	95 (44.2%)	64 (29.8%)	
26–35	315 (32.2%)	54 (17.1%)	166 (52.7%)	95 (30.2%)	
14–25	124 (12.7%)	18 (14.5%)	64 (51.6%)	42 (33.9%)	
Educational level					**<0.001**
No education	113 (11.6%)	22 (19.5%)	67 (59.3%)	24 (21.2%)	
1–5 years	526 (53.8%)	119 (22.6%)	280 (53.2%)	127 (24.1%)	
6–10 years	289 (29.6%)	49 (17%)	141 (48.8%)	99 (34.3%)	
11+ years	49 (5%)	14 (28.6%)	13 (26.5%)	22 (44.9%)	
Main source of family income					**0.035**
Agriculture	114 (11.7%)	26 (22.8%)	54 (47.4%)	34 (29.8%)	
Business	184 (18.8%)	43 (23.4%)	78 (42.4%)	63 (34.2%)	
Public service	65 (6.7%)	16 (24.6%)	26 (40%)	23 (35.4%)	
Day labor	353 (36.1%)	75 (21.2%)	196 (55.5%)	82 (23.2%)	
Fishing	190 (19.4%)	30 (15.8%)	112 (58.9%)	48 (25.3%)	
Others	71 (7.3%)	14 (19.7%)	35 (49.3%)	22 (31%)	
Main source of drinking water					**<0.001**
Tube well	211 (22.2%)	62 (29.4%)	98 (46.4%)	51 (24.2%)	
Pond/river/lake	103 (10.9%)	49 (47.6%)	28 (27.2%)	26 (25.2%)	
Supply	635 (66.9%)	88 (13.9%)	367 (57.8%)	180 (28.3%)	
Toilet facility					0.157
Slab toilet	858 (89.6%)	183 (21.3%)	436 (50.8%)	239 (27.9%)	
Open latrine	35 (3.7%)	5 (14.3%)	25 (71.4%)	5 (14.3%)	
Modern toilet	65 (6.8%)	12 (18.5%)	32 (49.2%)	21 (32.3%)	
BMI					**0.005**
Underweight	243 (25%)	54 (22.2%)	132 (54.3%)	57 (23.5%)	
Normal weight	575 (59.2%)	104 (18.1%)	292 (50.8%)	179 (31.1%)	
Overweight	103 (10.6%)	26 (25.2%)	50 (48.5%)	27 (26.2%)	
Obesity	51 (5.2%)	20 (39.2%)	22 (43.1%)	9 (17.6%)	
Level of physical labor					0.088
Very high	351 (35.9%)	71 (20.2%)	183 (52.1%)	97 (27.6%)	
High	274 (28%)	44 (16.1%)	141 (51.5%)	89 (32.5%)	
So-so	320 (32.8%)	79 (24.7%)	163 (50.9%)	78 (24.4%)	
Low	32 (3.3%)	10 (31.1%)	14 (43.8%)	8 (25%)	
Smoking status					0.233
Yes	164 (16.8%)	27 (16.5%)	85 (51.8%)	52 (31.7%)	
No	813 (82.3%)	177 (21.8%)	416 (51.2%)	220 (27.1%)	

Bold *p* values mean statistically significant.

a Total frequency is not always 977 due to missing or small frequencies in some categories (excluded).

The outcome variable revealed that the majority of the samples did suffer from a disease 1 month prior to the survey, although most of them belonged to the moderate group. In total, 20.9% went to a hospital/clinic for treatment of a disease 1 month prior to the survey and were, therefore, categorized as suffering from a severe disease. Furthermore, 51.3% reported a moderate disease since they sought treatment, if any, at other places except hospital/clinic, whereas only 27.8% mentioned that they did not have any disease in the past month prior to the survey.

### Simple and bivariable results

In the bivariable analysis, most of the basic variables except type of toilet facility, level of physical labor, and smoking status were significantly associated with the dependent variable (Table 1). These variables were later used in the multivariable analysis.

For factors reflecting the harm of livelihood due to EWCE consequences, most interviewees reported that their families have been harmed by salinization and river erosion (Table 2). All variables were highly significant in the bivariable analysis.

**Table 2:** Livelihood factors harmed by EWCE and their bivariable associations with severity of disease

Simple analysis		Severity of disease			
	
Variable	Frequency^a^	Severe disease	Moderate disease	No disease	*p*
Harm by salinity in land					**0.011**
Yes	707 (72.5%)	132 (18.7%)	375 (53%)	200 (28.3%)	
Moderately yes	146 (15%)	46 (31.5%)	67 (45.9%)	33 (22.6%)	
No	122 (12.5%)	25 (20.5%)	59 (48.4%)	38 (31.1%)	
Harm by river erosion					**<0.001**
Yes	610 (62.4%)	112 (18.4%)	330 (54.1%)	168 (27.5%)	
Moderately yes	114 (11.7%)	40 (35.1%)	60 (52.6%)	14 (12.3%)	
No	253 (25.9%)	52 (20.6%)	111 (43.9%)	90 (35.6%)	
Food scarcity in the past year					**<0.001**
Yes	452 (46.3%)	62 (13.7%)	284 (62.8%)	106 (23.5%)	
Moderately yes	222 (22.7%)	71 (32%)	94 (42.3%)	57 (25.7%)	
No	303 (31%)	71 (23.4%)	123 (40.6%)	109 (36%)	

Bold *p* values mean statistically significant.

a Total frequency is not always 977 due to missing or small frequencies in some categories (excluded).

Respondents’ perceptions of changes because of EWCE that reflect indirect consequences in their environment are presented in Table 3. Here, most of the samples mentioned a worsened situation regarding the loss of agricultural fields, employment problems, and drinking water availability resulting from EWCE. Similar results were found for water logging and loss of houses and animals, although to a smaller extent. In total, the majority of the study sample mentioned that most of the situations have worsened, with only few exceptions such as waterborne diseases and sanitation problems. Except two perception-related variables, namely ‘loss of agricultural fields’ and ‘water sewerage problems’, all other variables were significantly associated with the dependent variable in the bivariable analysis.

**Table 3:** Perception of changes in the livelihood factors due to EWCE-related consequences and their bivariable associations with severity of disease

Simple analysis		Severity of disease			
	
Variable	Frequency^a^	Severe disease	Moderate disease	No disease	*p*
Waterborne diseases					**0.003**
Increased	325 (33.3%)	55 (16.9%)	194 (59.7%)	76 (23.4%)	
Decreased	429 (44%)	97 (22.6%)	195 (45.5%)	137 (31.9%)	
Almost same	221 (22.7%)	52 (23.5%)	111 (50.2%)	58 (26.2%)	
Water logging					**0.019**
Increased	502 (51.5%)	125 (24.9%)	248 (49.4%)	129 (25.7%)	
Decreased	143 (14.7%)	26 (18.2%)	70 (49%)	47 (32.9%)	
Almost same	330 (33.8%)	53 (16.1%)	183 (55.5%)	94 (28.5%)	
Loss of houses and animals					**0.018**
Increased	536 (55.3%)	98 (18.3%)	271 (50.6%)	167 (31.2%)	
Decreased	173 (17.8%)	39 (22.5%)	99 (57.2%)	35 (20.2%)	
Almost same	261 (26.9%)	66 (25.3%)	126 (48.3%)	69 (26.4%)	
Loss of agricultural fields					0.68
Increased	627 (65%)	124 (19.8%)	329 (52.5%)	174 (27.8%)	
Decreased	106 (11%)	23 (21.7%)	53 (50%)	30 (28.3%)	
Almost same	232 (24%)	56 (24.1%)	111 (47.8%)	65 (28%)	
Social problems					**0.002**
Increased	398 (41%)	75 (18.8%)	226 (56.8%)	97 (24.4%)	
Decreased	374 (38.5%)	70 (18.7%)	185 (49.5%)	119 (31.8%)	
Almost same	199 (20.5%)	58 (29.1%)	86 (43.2%)	55 (27.6%)	
Water sewage problems					0.455
Increased	300 (31.3%)	72 (24%)	153 (51%)	75 (25%)	
Decreased	203 (21.1%)	43 (21.2%)	100 (49.3%)	60 (29.6%)	
Almost same	461 (47.8%)	88 (19.1%)	238 (51.6%)	135 (29.3%)	
Drinking water availability					**0.003**
Increased	214 (21.9%)	60 (28%)	112 (52.3%)	42 (19.6%)	
Decreased	590 (60.4%)	104 (17.6%)	307 (52%)	179 (30.3%)	
Almost same	173 (17.7%)	40 (23.1%)	82 (47.4%)	51 (29.5%)	
Employment problems					**<0.001**
Increased	600 (62.4%)	154 (25.7%)	281 (46.8%)	165 (27.5%)	
Decreased	183 (19%)	22 (12%)	99 (54.1%)	62 (33.9%)	
Almost same	178 (18.5%)	26 (14.6%)	110 (61.8%)	42 (23.6%)	
Sanitation problems					**<0.001**
Increased	117 (12%)	29 (24.8%)	67 (57.3%)	21 (17.9%)	
Decreased	574 (58.9%)	120 (20.9%)	265 (46.2%)	189 (32.9%)	
Almost same	284 (29.1%)	54 (19%)	168 (59.2%)	62 (21.8%)	

Bold *p* values mean statistically significant.

a Total frequency is not always 977 due to missing or small frequencies in some categories (excluded).

### Multivariable results

Multivariable multinomial logistic regression was performed using 16 independent variables. Possible confounding variables were forced into the model, as described in the methods section. Of the 16 variables, 10 variables still showed significant associations with severe diseases, moderate diseases, or both, as compared to no diseases (reference category). The resulting odds ratios (ORs) and associated confidence intervals (CIs) are displayed in Table 4 for severe diseases and in Table 5 for moderate diseases. For severe diseases, it was found that respondents who used drinking water from comparably unsafe sources, such as tube wells, or open sources, such as ponds, rivers, or lakes, had higher chances for severe diseases than those who mainly used supply water (OR: 2.39, 95% CI: 1.43–4.01 and OR: 4.26, 95% CI: 2.25–8.09, respectively). In addition, being moderately harmed by river erosion and food scarcity (OR: 6.24, 95% CI: 2.76–14.11 and OR: 1.98, 95% CI: 1.16–3.4, respectively), as well as a perceived increase of employment problems (OR: 2.19, 95% CI: 1.18–4.07) seemed to have a significant association with severe diseases. With respect to the confounding variables, high age and being female were associated with severe diseases, whereas the main source of family income, educational level, BMI, sanitation problems, and loss of houses and animals showed no significant association with severe diseases.

**Table 4:** Odds ratio (OR) and 95% confidence interval (95% CI) for severe diseases based on multivariable multinomial logistic regression (backward stepwise elimination method)

Associated factors	OR	95% CI	
Age			
56 +	4.14**	1.68	10.25
46–55	2.21	0.95	5.16
36–45	1.96	0.88	4.37
26–35	1.20	0.57	2.55
14–25	1		
Sex			
Female	3.46***	2.13	5.60
Male	1		
Main source of drinking water			
Tube well	2.39**	1.43	4.01
Pond/river/lake	4.26***	2.25	8.09
Supply water	1		
Food scarcity in the past year			
Yes	0.86	0.51	1.46
Moderately yes	1.98*	1.16	3.40
No	1		
Harm by river erosion			
Yes	1.34	0.82	2.20
Moderately yes	6.24***	2.76	14.11
No	1		
Employment problems			
Increased	2.19*	1.18	4.07
Decreased	0.82	0.38	1.78
Almost same	1		

*** *p*<0.001;

** *p*<0.01;

* *p*<0.05. Five variables were deleted automatically by backward stepwise procedure, 5 not shown due to insignificance.

**Table 5:** Odds ratio (OR) and 95% confidence interval (95% CI) for moderate diseases based on multivariable multinomial logistic regression (backward stepwise elimination method)

Associated factors	OR	95% CI	
Main source of family income			
Agriculture	1.14	0.51	2.53
Business	1.42	0.67	3.00
Service	0.98	0.39	2.45
Day labor	2.06*	1.04	4.10
Fishing	2.12*	1.01	4.45
Others	1		
Age			
56 +	3.47**	1.71	7.03
46–55	1.45	0.75	2.78
36–45	1.26	0.68	2.34
26–35	1.04	0.60	1.80
14–25	1		
Sex			
Female	2.60***	1.76	3.84
Male	1		
Educational level			
No education	2.42	0.91	6.43
1–5 years	2.36*	1.01	5.51
6–10 years	1.95	0.84	4.51
11+ years	1		
Sanitation problems			
Increased	1.79	0.92	3.50
Decreased	0.56**	0.37	0.84
Almost same	1		
Loss of houses and animals			
Increased	0.92	0.61	1.38
Decreased	1.79*	1.04	3.10
Almost same	1		
Food scarcity in the past year			
Yes	1.75**	1.16	2.63
Moderately yes	1.32	0.83	2.11
No	1		
Harm by river erosion			
Yes	1.3	0.87	1.94
Moderately yes	2.65**	1.28	5.48
No	1		
Employment problems			
Increased	0.70	0.44	1.12
Decreased	0.47**	0.27	0.81
Almost same	1		

*** *p*<0.001;

** *p*<0.01;

* *p*<0.05. Five variables were deleted automatically by backward stepwise procedure, 2 not shown due to insignificance.

For moderate diseases, more variables showed a significant association. Two categories for the main source of family income, namely day labor and fishing, were positively associated with higher ORs for moderate diseases, although the effect was rather weak. Similarly, respondents who completed 1–5 years of education had slightly higher OR for moderate diseases than those who completed 11 years or more. Notable results were found for the perceived decrease in problems of sanitation and employment, where the ORs were significantly smaller than compared with those who reported unchanged conditions for these incidences (OR: 0.56, 95% CI: 0.37–0.84 and OR: 0.47, 95% CI: 0.27–0.81, respectively). The only two variables which became insignificant were BMI and the main source of drinking water. The oldest age group, women, and those who were moderately harmed by river erosion had higher chances for moderate diseases. A fully experienced food scarcity was significantly associated with moderate diseases (OR: 1.75, 95% CI: 1.16–2.63).

## Discussion

The majority of our sample consisted of middle-aged people with moderate education who received most of their family income through day labor or by work in the primary sector. Most of them reported an increase of water logging, loss of houses and animals and agricultural fields, as well as an increase in employment problems. In addition, many were seriously harmed by river erosion and salinization. Only few people reported that they did not experience any disease 1 month prior to the survey, while it was found that severe diseases for which treatment was sought in hospitals/clinics were significantly associated with being female, higher age, drinking water from tube wells or open water sources, perception of increased employment problems, and being moderately harmed by both food scarcity and river erosion. Moderate diseases were significantly associated with perceived decrease in loss of houses and animals and employment problems, fully experienced food scarcity, being female, higher age, and moderate harm by river erosion. Furthermore, those who mainly relied on income acquired from day labor or fishing and those who completed 1–5 years of education also had a higher chance for moderate diseases.

A quite striking result was that only a few in the sample did not suffer from any disease. Moreover, the share of those who did not report any disease was only slightly different from those who were categorized as reporting severe diseases because of their hospital visit, while more than half suffered from moderate diseases according to our outcome categorization. This result is likely to be due to the nature of self-reported data. Especially those who were categorized as suffering from moderate diseases might have had health problems with no real clinical relevance, such as general malaise or diseases which lasted only for a short time. However, a similar consideration may also apply to hospital-based treatment, since a hospital visit might have been precautionary or might have turned out to be unnecessary.

For the analysis of possible associations with physical health, we chose factors which can be linked to EWCE and their consequences as well as important household characteristics that could influence health. As one surprising example regarding household characteristics, we found that most of the respondents’ main source of drinking water was supply water, although households in coastal Bangladesh usually rely on water from tube wells or open sources (18). Possible explanations for the high share of supply water may be the vast presence of non-government organizations (NGOs) in coastal Bangladesh (based on field experience) or vanishing sources of natural freshwater. We further examined the harm of livelihood by focusing on incidents such as salinity in land, river erosion, and food scarcity as possible consequences of EWCE. For all these three factors, the majority reported that they, including their respective families, experienced these problems and were directly harmed. Salinization can easily be linked to coastal flooding, a problem which is expected to increase against the background of climate change. It can also be linked with the reported increase in loss of agricultural fields, which in turn can lead to food scarcity, while further crop damage and food shortages caused by other EWCE is also possible (19). However, the harm of livelihood by salinization showed no significant association with the outcome, so that at least for this sample the effect on health can be neglected. Regarding the perception of EWCE-related consequences, the majority reported a worsening of most conditions that were mentioned in the questionnaire. Especially water logging, loss of houses and animals, loss of agricultural fields, a decrease in availability of drinking water, and employment problems seem to pose big challenges in the study area. By this descriptive analysis alone, the first links to indirect consequences on health can be drawn, while the reported problems also support the findings and suggestions of other studies (8, 10, 20). Although the perceived decrease in waterborne diseases was contrary to our expectations, a possible explanation could be specific interventions of NGOs, which can also be linked to the improved situation regarding sanitation facilities (based on field experience).

In the multivariable analysis, our findings showed significantly increased chances for severe and moderate diseases especially for typical risk groups such as women or the elderly, as well as higher chances for unsafe sources of drinking water, food scarcity, and river erosion. The association of severe diseases with the use of water from tube wells and ponds, rivers, or lakes for the purpose of drinking supports the hypothesis of increased health risks from the possible contamination of these water sources. As it was mentioned in the state of research, especially waterborne diseases are likely to be transmitted more easily in the course of advancing climate change, while the case of Bangladesh reveals particular vulnerability to flooding and thus to all possible consequences flooding brings along (8, 10, 16–18). On the contrary, we rather expected extensive harm by both river erosion and food scarcity to be associated with severe diseases. This finding could again be explained by the fact that our data reflect personal perceptions, so that an actual comprehension of the extent of a problem was not possible. The last associated factor with severe diseases, the increase of employment problems, may reduce the ability to afford for basic needs because of the loss of income, thereby hampering the improvement in the general well-being of the populace, leading to health problems.

A positive association of the main source of family income was found only with moderate diseases. Here, the two income sources, day labor and fishing, revealed higher ORs for moderate diseases, although the effect was quite small. However, especially since day labor can undoubtedly be seen as the most insecure source of income generation, it can be assumed that the livelihood of a household that relied on day labor was very unstable and, thus, more vulnerable to possible health risks. The lower OR for moderate diseases for those who reported an actual decrease in employment problems may further support this suggestion. Similarly, the lower OR for reported improvements of sanitary conditions can be seen as a sign for improved health and specific interventions of NGOs. The association of fully experienced food scarcity and moderate diseases, which was actually expected for severe diseases, might indicate that food scarcity was not an enduring problem for our study sample. As a consequence, more serious incidents such as malnutrition and possibly resulting diseases can be ruled out, although these are still big public health challenges in Bangladesh (22). The last result for moderate diseases, the positive association with a perceived decrease of loss of houses and animals, is quite difficult to explain, although the association was also rather weak. One possible explanation could be the role of post-hazard management, where those households who actually were harmed by loss of houses and animals might have received more help in the aftermath of an EWCE, while those who reported a decrease might not have been harmed and, thus, were not considered for certain aid measures. However, this result is subject to speculation.

Our study has several strengths and limitations. Only few empirical studies have been conducted with the aim of identifying EWCE-related consequences on health, although it is known that Bangladesh is one of the most natural disaster–prone countries. Therefore, our study helps to quantify problems which are actually well-known but are still lacking empirical data. Although we did not focus on a particular disease, we included several factors that can be associated with EWCE, especially with indirect consequences. Most findings thereby correspond to those found in other studies. A further strength is that we focused on the perception of the survey respondents. In our view, the inclusion of perceptions and opinions of the population at risk reflects valuable information to complete the picture of climate change–related challenges. On this basis, evidence-based and needs-assessed intervention strategies can be formulated. However, the perception of EWCE-related consequences by the sample is also one of the study's limitations. As indicated, it cannot be assured that the respective respondent or his or her household was actually harmed by certain events. Some indicators, such as social problems and the loss of agricultural fields, might have been visible in the respondents’ environment, but the respective respondents might not have been harmed themselves. For instance, families that mainly rely on income acquired through service work might perceive an increased damage of crop yields or livestock in the neighborhood, but these incidents might have been less impairing than for those families who actually rely on these factors. Furthermore, the use of the main source of family income as a proxy for the socioeconomic status might not properly reflect the actual socioeconomic reality of a family. Although seasonal variations can skew the average annual income especially in the agricultural sector or for families relying on day labor, wealth disparities may still exist within groups of income sources. Another limitation already mentioned deals with the derivation of the outcome variable. Especially for health-related issues, self-reported data bear several problems: For our analysis, some doubts remain as to whether the categorization based on the respective source of treatment reflected the actual situation properly because objective, clinical confirmations were missing. Moreover, the source of treatment could be influenced by the socioeconomic status or by the lack of health knowledge, leading to potential difficulties and disadvantages regarding access to health care.

## Conclusions

Given advancing climate change, the consequences of EWCE for Bangladesh can be expected to worsen. Especially, indirect consequences which are present in the long run after any EWCE event are likely to widen the range of possible adverse health outcomes. Physical health can especially be threatened by contaminated drinking water, which in turn can be linked to flooding and associated consequences such as damage of the sewage system, influencing the spread of infectious diseases. Furthermore, EWCE have an impact on food production and are likely to cause food insecurity due to the damage of crop yields, whereas salinization of agricultural fields after flooding can be seen as a typical example of indirect EWCE consequences, which render agricultural fields useless. In summary, particularly the impact of EWCE on basic needs, such as drinking water and food, can turn into ill-health, calling for intervention strategies which protect freshwater flows, supply more clean drinking water, and secure the food production and distribution system. Interventions addressing education and awareness raising should also be strengthened, although a lot has been achieved in the recent years (10, 23). However, more research is needed with the aim of identifying concrete health risks and their impact on specific diseases as influencing factors. Future studies should, therefore, be conducted in a longitudinal or case–control design to reveal causal relationships and to contribute to scarce empirical data.
